# Deep Brain Stimulation in Tourette’s Syndrome

**DOI:** 10.3389/fneur.2015.00170

**Published:** 2015-08-04

**Authors:** Avram Fraint, Gian Pal

**Affiliations:** ^1^Department of Neurological Sciences, Rush University Medical Center, Chicago, IL, USA

**Keywords:** deep brain stimulation, DBS, Tourette’s syndrome, tics, TS

## Abstract

**Objective:**

Tourette’s syndrome (TS) is defined by 1 year of persistent motor and vocal tics. Often, the tics are refractory to conventional pharmacologic and psychobehavioral interventions. In these patients, deep brain stimulation (DBS) may be an appropriate intervention. This paper reviews different DBS targets in TS, discusses existing evidence on the efficacy of DBS in TS, highlights adverse effects of the procedure, discusses indications and patient selection as well as future directions for DBS in TS.

**Methods:**

A literature review searching PubMed database entries between 2000 and 2015. Search terms included “DBS in Tourette Syndrome”, “Deep brain stimulation in Tourette syndrome,” and “Surgical management of Tourette Syndrome.”

**Results:**

Though there are no universally accepted guidelines defining ideal DBS candidates for TS, age, tic severity, and treatment refractoriness are important factors to consider in patient selection. A variety of targets exist for DBS in TS, but thalamic targets and GPi are the most widely studied. Psychiatric side effects that are target specific should be monitored closely and it is possible that these adverse effects may be resolved with programing. Small randomized controlled trials support the efficacy of DBS in TS.

**Conclusion:**

DBS for TS is safe and feasible, but large multi-center clinical trials are needed to determine the ideal target and optimal location within a particular target.

## Introduction

Tourette’s syndrome (TS) is defined by 1 year of persistent, waxing and waning motor, and vocal tics. Up to 90% of patients with TS have co-morbid psychiatric conditions including Obsessive-Compulsive Disorder (OCD) and Attention Deficit Hyperactivity Disorder (ADHD). Tics usually occur at a mean age of 5–7 years and usually remit by early adulthood (1). Patients with TS usually respond well to pharmacologic treatments, including alpha_2_ adrenergics, typical and atypical neuroleptics, and ADHD medications. Psychobehavioral therapies, including habit reversal therapy (HRT), are also integral to TS treatment. Despite the availability of numerous treatments, some cases remain refractory to pharmacologic and behavioral interventions.

Deep brain stimulation (DBS) may be an option for medically intractable and severe cases of TS. Very severe tics and psychiatric co-morbidities can cause social impairment, isolation, and pain, and in these patients DBS may be appropriate. Though DBS has shown clear efficacy in other movement disorders including Parkinson’s disease (PD), dystonia, and essential tremor (ET), the benefit of DBS in TS is still unclear for a variety of reasons. First, no consensus has been reached concerning which TS patients are appropriate candidates for DBS. Second, the pathophysiology of the disease remains to be elucidated and hence the optimal target has yet to be identified. Further, many of the studies of DBS in TS are only case reports and not randomized controlled trials (RCTs). Lastly, the population of TS patients is not homogenous, and since waxing and waning symptoms are part of the disease itself, it is difficult in these cases to truly assess the long-term benefit of DBS.

We aim to shed light on these issues by highlighting the most researched targets for DBS in TS, reviewing indications and patient selection, and describing adverse effects through the lens of the most recent and highest quality level of evidence available to date.

## Methods

We reviewed PubMed entries between 2000 and 2015. The following search terms were used: “DBS in Tourette Syndrome,” “Deep brain stimulation in Tourette syndrome,” and “Surgical management of Tourette Syndrome.” The search engine generated 201 publications. Epidemiologic studies, case series, and RCTs were included and reviewed. All journal articles reviewed were written in English.

## Current Indications and Patient Selection

There are no universally accepted guidelines defining ideal DBS candidates for TS. Indeed, many different groups have proposed slightly different guidelines over time, but no single guideline has gained widespread acceptance (2, 3). However, common themes can be gleaned from the available guidelines, which are summarized in Table 1. First, the patient must be diagnosed with TS according to the criteria specified in the DSM-5 and by a clinician who has experience with the diagnosis of TS and tic disorders. Tics should be the main symptom and they must cause significant impairment in quality of life, particularly impacting relationships, home environment and/or school/work. The tics should be treatment resistant and co-morbid psychiatric diagnoses must be adequately treated to maximize follow up and compliance. The patient should not suffer from behaviors that will lead to damage of the electrodes or stimulator, including obsessive picking at the insertion site so as to reduce the risk of infection (4). Ultimately, three main factors influence patient selection for DBS in TS – age, tic severity, and treatment refractoriness.

**Table 1:** **Proposed inclusion and exclusion criteria for DBS use in TS**.

Inclusion criteria	Exclusion criteria
At least 18 years old. Younger patients would require approval from local ethics committee	Under 18 years old without approval from local ethics committee
DSM-V diagnosis of TS	Active suicidal or homicidal ideation
Severe tics, as defined by YGTSS >35/50	Ongoing or recent substance abuse
Tics are the main source of disability	Structural lesions on MRI
Tics are refractory to three classes of conservative pharmacologic therapy and CBT has been offered	Co-morbid medical or psychiatric conditions that increase the risk of a failed procedure or interference with post-operative management
Psychiatric co-morbidities are being treated and are stable for at least 6 months	Malingering, factitious, psychogenictics
Stable environment with reliable and stable social supports	
Demonstrated adherence to recommended therapies	
Neuropsychological profile indicating the patient can tolerate demands of surgery and post-operative follow up schedule	

It is generally accepted that DBS should be reserved for adults, as most cases of TS remit spontaneously during early adulthood (5). Typically, guidelines have recommended offering the surgery only to individuals who are at least 18 years of age, but others recommend a minimum cutoff of 25 (6). The youngest reported patient who has undergone DBS for TS is 16, however, this patient was unique in that he was mentally disabled and his disease was so severe and refractory to typical treatments that he became depressed, isolated, and suicidal (7). Most recently, Schrock et al. indicated that age alone is not a strict exclusion criterion for DBS in TS (2015), however, that for patients under 18, a local ethics committee should be consulted.

There is agreement that only patients with “severe” TS should be considered candidates for DBS (4, 8). Scales such as the Yale Global Tic Severity Scale (YGTSS) are generally used to quantify disease severity (9). Most guidelines suggest that patients should have a score ≥35/50 on the YGTSS, which is generally accepted as “severe.” However, others argue that an assessment of social impairment is required to truly assess disease severity, and this is a limitation of using the YGTSS alone (1). Further, premonitory sensations, somatic aspects of the disease and self-perception of severity may also be important factors to consider when assessing severity, rather than relying on strict cut-off scores (1). Schrock et al. (3) propose that the tics should cause functional impairment, but agree that YGTSS above 35/50 defines “severe” disease.

It is accepted that DBS candidates’ disease must be “resistant” or “refractory” to pharmacologic and behavioral treatments (4), but the definition of these terms is variable. According to the TSA guidelines (10), TS patients should have failed treatment with or had severe side effects from alpha-adrenergic agonists, typical and atypical antipsychotics and a benzodiazepine. The Dutch/Flemish guidelines (11) suggest that at least three medications (including typical and atypical antipsychotics) should be tried at adequate doses for 12 weeks while other groups have suggested a minimum treatment period of 6 months (1) to be considered medication-refractory. The European Society for Study of Tourette Syndrome (ESSTS) guidelines suggest that the tics must be present for at least 5 years and must be considered “severe” for 1 year before DBS is performed (4). Schrock et al. (3) require failed treatment trials of alpha-adrenergic agonists, dopamine antagonists (both typical and atypical), and a drug from a third medication class.

Most guidelines also recommend trial and failure of behavioral therapy before considering DBS. According to the TSA guidelines, patients must have failed 12 successive sessions of behavioral therapy, including habit reversal and exposure type therapies. Schrock et al. indicate that an expert clinician should be comfortable ruling out malingering, factitious disorder and psychogenic tics before proceeding with surgery and selected patients should be capable of adhering to proposed follow up guidelines. Schrock et al. propose that a trial of CBT should be offered. They add that adequate social support should be evident with a caregiver who is available to accompany the patient to frequent follow-up visits (2015).

## DBS Targets for TS

**Figure 1:**
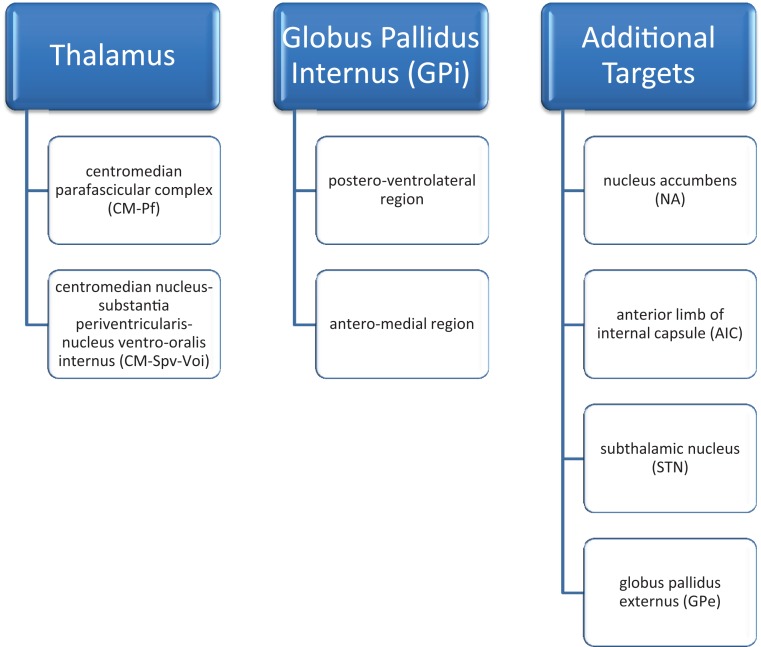
**DBS targets in Tourette’s syndrome [Servello et al. (12); Servello et al. (13); Shields et al. (14)]**.

At the present time, no definitive surgical target has been agreed upon. Due to wide inter-patient variability and co-morbidities, multiple targets have been used. A total of eight suitable targets have been identified: two in the thalamus [the centromedian parafascicular complex (CM–Pf) and centromedian nucleus-substantia periventricularis-nucleus ventro-oralis internus (CM–Spv–Voi)], two in the globus pallidus internus (the postero-ventrolateral region and the antero-medial region), the nucleus accumbens (NA), the anterior limb of the internal capsule (AIC), the subthalamic nucleus (STN) and the globus pallidus externus (GPe). Combinations of these different targets have been tried as well (12–14) (Figure 1). Despite the variety and combinations tried, the thalamus and globus pallidus internus are the most widely studied and targeted areas for TS.

### Thalamic targets

The CM–Pf region of the thalamus is by far the most widely studied target, as cells in its anterior region are thought to influence tic generation (1). In 1970, Hassler and Dieckmann described three patients who underwent thalamotomy of the CM–Pf for treatment of TS (15). One patient reported improvement in echolalia within hours after surgery and complete remission of premonitory urges toward tics as well as coprolalia when followed up 1 year later. The second patient reported complete remission of coprolalia soon after surgery, and the third reported a reduction in tics and obsessive crying by 1 year after surgery. However, it was not until 1999 that Visser-Vandewalle et al. used thalamic DBS to treat severe and refractory TS in a 42-year-old man. They reported substantial improvement in frequency of motor tics at a 4 month post-operative time point (16). Additional larger series have also reported significant improvement with thalamic targeting. Servello et al. (12) examined the effects of CM–Pf stimulation in 34 subjects. The average YGTSS score improved from 75.5 ± 12.6 to 40.0 ± 14.1 (*p* < 0.001) over a follow-up period of 3 months to 2 years. Similarly, Porta et al. (1) described a series of 15 patients who experienced an improvement of mean YGTSS from 76.5 to 36.6 at 2-year follow up. Symptoms of depression, anxiety, OCD, obsessive-compulsive behaviors (OCBs), and subjective perception of social impairment decreased as well. Specifically, Yale-Brown Obsessive Compulsive Scale (YBOCS) scores improved from 20.9 to 14.4 (*p* = 0.009), Beck Depression Inventory (BDI) scores improved from 30.7 to 22.7 (*p* = 0.001) and State-Trait Anxiety Inventory (STAI) scores improved from 44.2 to 29.5 (*p* = 0.001).

Another region of the thalamus, the CM–Spv–Voi was shown to be effective in three adults for medically intractable TS (17). However, no systematic scale was used to evaluate these patients, but rather improvement was based on number of tics seen during a specified time period at a particular stimulation level.

The efficacy of thalamic DBS for TS has been attributed to modulation of excessive thalamocortical drive (18). It has been posited that cortical excitation of the striatum and STN results in inhibitory projections to the thalamus and midbrain, thus modulating motor patterns of the cerebral cortex and brainstem (18). Further, abnormal inhibition of the GPi and substantia nigra pars reticulata (SNpr) by inappropriate activation of striatal neurons may lead to increased thalamocortical drive, resulting in unwanted motor patterns and execution of tics (18). There is also evidence for thalamic DBS resulting in a reduction of increased dopaminergic transmission in the thalamus (19, 20).

### Globus pallidus internus

Globus pallidus internus DBS has been explored as another key target for TS DBS. Cavanna et al. (6) cite four key reasons for GPi as their preferred target for TS: (1) GPi plays a central role in the cortico-striato-thalamo-cortical circuitry involved in TS pathophysiology; (2) GPi has been shown to be more effective than thalamic DBS in one small series (21); (3) lack of side effects with GPi stimulation; (4) GPi is readily visible on MRI for more facile implantation.

In a small pilot study, Welter et al. (21) found bilateral GPi stimulation produced a more favorable outcome in tic severity (78% reduction) compared with thalamic CM–Pfc stimulation (45% reduction). Larger series also support the efficacy of GPi stimulation for TS. Cannon et al. (22) reported on eleven patients that had GPi DBS implantation for TS and these subjects had a mean reduction of 50% in the YGTSS at 3 months of follow up. Similarly, Zhang et al. (23) reported outcomes in 13 subjects with treatment refractory TS who underwent GPi DBS. These subjects had a mean reduction in YGTSS of 52.1% (range 4.3–83.6%) over a mean follow-up period of 41.9 months (range 13–80 months). Also, the Gilles de la Tourette Syndrome-Quality of Life Scale score improved by a mean of 45.7% (range 11.0–77.2%).

Though GPi is a promising target, there is a debate regarding the optimal location for electrode placement in GPi. Some groups have suggested the posteroventral (sensorimotor) region (24–27), while others have targeted the antero-medial (limbic) portion of GPi (21, 28, 29). Large multi-center trials are needed to determine the efficacy of DBS in GPi, as well as the optimal location within GPi for stimulation.

### Other targets

In addition to the thalamus and GPi, other DBS targets have been explored as well. Sturm et al. (30) reported effectiveness of DBS of the NA in four patients with severe OCD and anxiety. Welter et al. (21) reported a significant and greater reduction in YGTSS with stimulation of the NA compared to stimulation of CM/Pf of the thalamus in three patients with severe TS. Kuhn et al. (31) reported that DBS of the NA resolved tics and coprolalia in one 26-year-old man with severe TS. Flaherty et al. (32) reported one woman who underwent DBS of the AIC (with an electrode terminating in the NA) and experienced significant reduction in tic frequency and severity at 18 months. Though these different targets have been explored, they are only used in small case series. The focus remains on the thalamus and GPi as the most promising potential targets.

## Randomized Controlled Trials Supporting the Efficacy of DBS for TS

At the present time, five randomized double-blind control trials have evaluated the utility of DBS for TS. Houeto et al. (29) reported a prospective, double-blind study of one patient who underwent bilateral CM–Pf thalamic and bilateral antero-medial GPi lead implantation. Tic severity was assessed 1 month before implantation and then at various intervals after surgery in a double-blinded, randomized protocol in five phases: no stimulation, bilateral thalamic stimulation, bilateral GPi stimulation, a sham trial where the stimulators were turned off, and combined thalamic and GPi stimulation. After bilateral thalamic stimulation, the patient had a 65% reduction in the YGTSS and a 77% improvement on the Rush Video-Based Tic Rating Scale (RVBTS) after 2 months of stimulation. The patient also reported fewer self-injurious behaviors. Bilateral GPi stimulation produced a 65% reduction in the YGTSS and a 67% improvement in the RVBTS after 2 months of stimulation. GPi stimulation also produced a reduction in self-injurious behaviors, though mood and impulsivity were worse compared to bilateral thalamic stimulation. Interestingly, 2 months of combined bilateral thalamic and GPi stimulation produced complete cessation of self-injurious behavior and a 70% reduction in YGTSS, and these improvements persisted at 2 years after the procedure.

Maciunas et al. (33) reported five patients who underwent stimulation of bilateral thalamic CM–Pf in a randomized, double-blinded trial. The randomization period began 1 month after implantation. The patients spent 1 week in each of the following states: both stimulators off, left on and right off, right on and left off, and both on. Blinded subjective and objective results were assessed at the end of each week. There was a statistically significant reduction of 4.2 points (a 53% reduction in tics) in the modified Rush Video-Based Tic Rating Scale (mRVBTS) score in the bilateral stimulation state. There was improvement in motor and vocal tics as well as in the YGTSS and TS Symptom List scores. Results were similarly assessed in an un-blinded fashion after 3 months of bilateral stimulation, which showed persistent benefit.

Welter et al. (21) reported a double-blind, randomized cross-over study on the effect of high frequency stimulation of the CM–Pf and/or the ventro-medial GPi which was mentioned briefly above. Three patients with severe TS were selected for electrode implantation. Patients were examined 1 month before surgery and 2 months after surgery without stimulation. Four stimulation conditions were randomly assigned in a cross-over design. The conditions were (1) bilateral thalamic stimulation, (2) bilateral GPi stimulation, (3) combined bilateral pallidal and thalamic stimulation and (4) no stimulation. Each stimulation condition was maintained for 2 months and patients were examined monthly by blinded clinicians. The study revealed improvement in the YGTSS with bilateral GPi stimulation (tic severity reduction ranged from 65 to 96%). Bilateral CM–Pf stimulation also reduced tic severity, but not as dramatically (range of reduction from 30 to 64%). Interestingly, combined GPi and thalamic stimulation did not show a further reduction in tic severity. Motor symptoms recurred during the sham stimulation and no neuropsychological, psychiatric or other long-term adverse effects were observed.

Ackermans et al. (34) performed a randomized double-blind cross-over study in six patients to assess safety of stimulation of the Cm–Spv–Voi in the thalamus. After surgery, the patients were randomly assigned to two groups: Group A had stimulators turned on during the first 3 months followed by 3 months with their stimulators off; Group B had the opposite stimulation schedule. This was followed by 6 months with the stimulators turned on in both groups. Assessments were performed before surgery and at 3, 6, and 12 months after surgery. Tic severity during the “on-stimulation” period was significantly lower than during the “off-stimulation” period, with 37% improvement in YGTSS (p = 0.046). There was a sustained effect of stimulation 1 year after surgery, with 49% improvement in YGTSS compared to pre-operative assessments (*p* = 0.028). Recently, Kefalopoulou published an RCT on pallidal DBS in TS, which indicated that bilateral GPi (both antero-medial and posteroventral regions) stimulation lead to improved tic severity, and was safe (see Table 2 for summary of RCTs).

**Table 2:** **Randomized controlled trials of DBS in TS**.

Study	Target	Sample size	Outcomes	Adverse effects
Houeto et al. (29)	CM–Pf + bilat GPi	1	Bilateral thalamic stimulation: 65% reduction in YGTSS, 77% improvement in RVBTS, fewer self-injurious behaviors; Bilateral GPi stimulation: 65% reduction in YGTSS, 67% improvement in RVCTS, fewer self-injurious behaviors, but mood and impulsivity worse compared to bilateral thalamic stimulation	Weight loss
Ackermans et al. (34)	CM–Spv–Voi	8	49% improvement in YGTSS, 35% improvement in RVTRS	Decreased energy, subjective visual disturbance, one small hemorrhage, persistent nystagmus
Maciunas et al. (33)	CM–Pf	5	40–67% reduction in RVTRS, 21–70% mean reduction in vocal tics, 43.6% reduction in YGTSS, 43% mean reduction in TSSL	Two patients had tic exacerbation, one patient had acute psychosis
Welter et al. (21)	CM–Pf + Gpi	3	65–95% improvement In YGTSS with GPi only, 30–64% improvement in YGTSS with CM–Pf only, 43–76% improvement in YGTSS with combined stimulation	Decreased libido with thalamic stimulation; lethargy, nausea and vertigo at high settings of GPi stimulation
Kefalopoulou et al. (35)	Bilat Gpi	15	Mean YGTSS scores were significantly lower at the end of the on-stimulation period (mean improvement 12.4, or 15.3%	Two infections of DBS hardware, one episode of hypomania. All resolved with treatment.

## Adverse Effects

Severe surgery-related adverse effects are rare. Overall estimates of the incidence of symptomatic intracerebral hemorrhage as a result of DBS for all indications are about 1% (36). Infection is another potential complication, particularly from *Staphylococcus aureus* in the infraclavicular region near the battery insertion site (34). If infection does occur, it must be identified and treated promptly, otherwise the DBS leads may need to be extracted. Reported complication rates are variable depending on the series. Intracranial complications such as infection, hemorrhage, ischemic events, and microelectrode rupture/displacement have been reported to occur in 3.2% of subjects (37). In another series, Servello et al. (38) reported that DBS-related infection rates were 18% in TS compared to 3.7% overall (including DBS for PD, dystonia and ET). They speculated that this might be due to compulsive touching of the scar which is common in patients with TS. Other complications including subcutaneous pouch-related complications such as seroma or hematoma, wound diasthesis or infection (pouch or extension cables) have to reported in 19.3% of subjects (37).

Changes in sexual behavior have been reported in subjects after DBS of the CM–Spv–Voi (17). Houeto et al. (29) noted stimulation-related weight loss after both thalamic and antero-medial GPi stimulation. This is interesting considering PD patients typically report weight gain after DBS (39). Reported psychiatric symptoms include psychosis (33), depression, and hypomania (32). Nausea, vertigo, anxiety, and social avoidance have been reported after ventromedial GPi stimulation (21, 24, 28). Welter et al. (21) reported transient oral or arm paresthesias with thalamic stimulation, while pallidal stimulation induced lethargy that lasted 3–4 days. Thalamic stimulation has also been associated with decreased libido (21). Zhang et al. (23) reported that patients with GPi stimulation experienced mood symptoms including anxiety and agitation. Interestingly, they reported that these side effects could be resolved with detailed programing.

Nucleus accumbens stimulation can result in adverse effects including flushing, anxiety, sweating, hypomania, agitation, and psychosis (40). Adverse effects of DBS of the AIC to treat OCBs and TS include euphoria, giddiness, anxiety, panic, fear, and acutely worsening depression (41).

## Future Directions

There are multiple studies ongoing investigating the use of DBS in TS. A randomized double-blind safety/efficacy study (Clinicaltrials.gov, NCT02112253) is being conducted to define the optimal location and stimulation settings for the anterior globus pallidus. The study examines deep versus superficial electrode contact positions, compares two different amplitudes of stimulation, and aims to recruit ten subjects. Primary outcome measures include YGTSS scores at baseline and 3, 6, 9, 12 and 18 months post-stimulation. Two studies (Clinicaltrials.gov NCT02056873 and NCT01817517) are being conducted to evaluate the effectiveness and safety of thalamic DBS in ten subjects. Certainly more work is needed to understand the full potential of DBS in TS.

It is clear that further RCTs are needed to accurately determine the effectiveness of DBS, and as a means of comparing one target to another. To do so, uniform outcome measures should be selected in the assessment of tic severity and reduction as well as quality of life measures. Of course, clinical scores of tic severity, such as the YGTSS, are inadequate by themselves, since they may not account for subjective feelings of isolation, and depression which accompany TS. It has been argued that clinician assessed video recordings, such as the RVBTS, may introduce measurement and error bias (42).

## Summary/Key Points

Reports thus far indicate that DBS is a safe and feasible therapy, but its efficacy for TS remains to be fully elucidated. Selecting TS patients for DBS is challenging given the unpredictable natural history of the disease, the varied extent to which TS patients are hampered by their tics, the presence of co-morbid psychiatric conditions, and the lack of consensus regarding implementation of this procedure. Additionally, multiple suitable targets have been identified, adding another level of complexity. Ultimately, each TS patient should be evaluated individually to determine their suitability for DBS. The above studies have shown that DBS for TS is relatively safe and feasible, but large multi-center clinical trials are needed to determine the ideal target for TS and optimal location within a particular target.

## Conflict of Interest Statement

The authors declare that the research was conducted in the absence of any commercial or financial relationships that could be construed as a potential conflict of interest.
